# A COUNTRY STORY

**DOI:** 10.3201/eid1311.079999

**Published:** 2007-11

**Authors:** Kenneth Fields

“When I was a little girl back in East Texas,”
My mother’s mother, Beulah, used to tell,
“There was an outbreak of the German measles,
Mama was pregnant, so I went away
To a neighbor lady’s, three or four miles from home
When the first signs showed. I was just eight, and sick,
And lonesome for Mama. One day she came for me.
My little sister had broken out, and Mama
Figuring she would die, and the baby, too,
Wanted us all together for those last weeks.
She wanted me home with her. As it turned out
My sister had been reading by the fire
And broke out from the heat, and it was me
That carried the measles home. After Mama died
I used to think of seeing her out the window
Talking to the neighbor lady on that day,
Crying and wiping her eyes with her apron hem.”

